# Effect of Leg Dominance on The Center-of-Mass Kinematics During an Inside-of-the-Foot Kick in Amateur Soccer Players

**DOI:** 10.2478/hukin-2014-0060

**Published:** 2014-10-10

**Authors:** Matteo Zago, Andrea Francesco Motta, Andrea Mapelli, Isabella Annoni, Christel Galvani, Chiarella Sforza

**Affiliations:** 1 Laboratory of Movement Analysis, Dipartimento di Scienze Biomediche per la Salute, Università degli Studi di Milano, Italy.; 2 Department of Bioengineering, Politecnico di Milano, Milano, Italy and Laboratory of Movement Analysis, Dipartimento di Scienze Biomediche per la Salute, Università degli Studi di Milano, Italy.; 3 Department of Psychology, Exercise and Sport Science Degree Course, Catholic University of the Sacred Heart, Milan, Italy.; 4 Dipartimento di Scienze Biomediche per la Salute, Facoltà di Medicina e Chirurgia, Università degli Studi di Milano, Italy.

**Keywords:** soccer biomechanics, laterality, joint angle, postural control, technical skills

## Abstract

Soccer kicking kinematics has received wide interest in literature. However, while the instep-kick has been broadly studied, only few researchers investigated the inside-of-the-foot kick, which is one of the most frequently performed techniques during games. In particular, little knowledge is available about differences in kinematics when kicking with the preferred and non-preferred leg. A motion analysis system recorded the three-dimensional coordinates of reflective markers placed upon the body of nine amateur soccer players (23.0 ± 2.1 years, BMI 22.2 ± 2.6 kg/m2), who performed 30 pass-kicks each, 15 with the preferred and 15 with the non-preferred leg. We investigated skill kinematics while maintaining a perspective on the complete picture of movement, looking for laterality related differences. The main focus was laid on: anatomical angles, contribution of upper limbs in kick biomechanics, kinematics of the body Center of Mass (CoM), which describes the whole body movement and is related to balance and stability. When kicking with the preferred leg, CoM displacement during the ground-support phase was 13% higher (p<0.001), normalized CoM height was 1.3% lower (p<0.001) and CoM velocity 10% higher (p<0.01); foot and shank velocities were about 5% higher (p<0.01); arms were more abducted (p<0.01); shoulders were rotated more towards the target (p<0.01, 6° mean orientation difference).

We concluded that differences in motor control between preferred and non-preferred leg kicks exist, particularly in the movement velocity and upper body kinematics. Coaches can use these results to provide effective instructions to players in the learning process, moving their focus on kicking speed and upper body behavior.

## Introduction

The inside-of-the-foot kick (pass-kick) can be considered as fundamental in soccer requiring both technical and tactical individual skills. Together with dribbling, a pass-kick is the most frequently performed technique during match play (Reilly et al., 2000): the ball is hit by the medial part of the foot, providing accuracy and precision (Nunome et al., 2006). For this reason, the inside-of-the-foot kick is the building block of combination play and collective tactics, and it is essential for retaining possession (O’Reilly and Wong, 2012).

Several studies have been conducted regarding kicking in soccer, and comprehensive knowledge about the three-dimensional kinematics and kinetics is available. However, the majority of studies were about the instep (full) kick (Dörge et al., 2002; Katis and Kellis, 2010; Katis et al., 2013; Kellis and Katis, 2007; Lees et al., 2010; Nunome et al., 2006) or about outside-of-the-foot kicking (Katis and Kellis, 2010; Kawamoto et al., 2007). Only Levanon and Dapena (1998) considered the inside-of-the-foot kick, comparing it to the instep-kick. Authors agreed that in both kicking techniques the kicking leg behaves as a three-link kinetic chain made up by thigh, shank and foot. They also described the phases of movement in detail: the approach run (i) ends when the support heel lands (heel-strike) on the ground. The backswing phase (ii): the hip is extended, slowly adducted and externally rotated, the knee flexed and internally rotated, while the ankle is plantar flexed and abducted. Forward motion: (iii) the pelvis is rotated around the support leg and the hip starts to flex and abducts while it remains externally rotated. Simultaneously, the ankle is plantar flexed, and knee extension velocity is maximized. Upon impact (iv) the hip is flexed, abducted and externally rotated and the ankle plantar flexed and adducted.

All the previous investigations concentrated on the lower limbs. Shan and Westerhoff (2005) introduced a total-body analysis based on the hypothesis that upper-body movement might be a key factor in creating the right conditions for an effective kick. They claimed that quick trunk flexion and rotation towards the kick side, accompanied by fast arm flexion and adduction on the non-kick side, contribute to an explosive muscle contraction and permit a powerful whip-like movement of the kicking leg. The study analyzed the instep-kick only; yet, the role of arms and upper-body kinematics in the inside-of-the-foot kick is still unknown.

Manolopoulos et al. (2006) explored the body Center of Mass (CoM) displacements throughout the kick phases. In soccer, CoM displacement and velocity are indicative of the player’s stability during the kick (Manolopoulos et al., 2006). Indeed, the movement of an individual’s CoM summarizes the whole body mass movement and has been used to investigate technique in many sports like running, volleyball (Wagner and Tilp, 2009), martial arts (Imamura et al., 2006), ice-skating (Mapelli et al., 2013). CoM kinematics can provide useful information about body balance, allowing to explore the level of performance and expertise of an athlete. In particular, CoM horizontal movement was associated with balance and stability (Halvorsen et al., 2009). Our hypothesis is that the analysis of the three-dimensional coordinates and velocity of the CoM can provide an interesting - and barely explored in soccer - perspective on the kinematics of a specific technique. This approach allows comparing traditionally studied kinematics determinants to the global characteristics of the movement, described by the CoM itself.

Laterality-related kinematic differences when performing an instep-kick with the preferred and non-preferred leg were found by Dörge et al. (2002), Nunome et al. (2006) and Teixeira and Teixeira (2008). When kicking with their preferred leg, players showed higher foot and shank velocities and better inter-segmental motion patterns. However, it is not clear if the same holds true for the inside-of-the-foot kick. Fletcher and Long (2013) observed that players were able to maintain better dynamic stability when kicking with the preferred leg. On the converse, when kicking with the non-preferred leg, thus when balancing on the preferred leg, they showed worse dynamic balance. However, no explanatory kinematic data were given. On the basis of field observations, we hypothesized that kinematic differences should exist between preferred and non-preferred leg inside-of-the-foot kicking. Knowing them may produce more specific and effective instructions for players in the training program.

The purpose of this study therefore was two-fold: 1) to apply a total-body, CoM-based approach to the analysis of the kinematics of the inside-of-the-foot kick in soccer, and 2) to assess which differences, if any, arise when performing an inside-of-the-foot kick with the preferred and the non-preferred leg. Based on the assessed literature, we expected 1) better skill proficiency when kicking with the preferred leg, faster CoM movements and more coordinate use of upper body; 2) higher kicking leg segments velocities, possibly produced by wider hip flexion and knee extension, when kicking with the preferred leg.

## Material and Methods

### Participants and procedures

Nine amateur male soccer players (23.0 ± 2.1 years, body height 174.0 ± 3.4 cm; body mass 67.2 ± 8.1 kg, BMI 22.2 ± 2.6 kg/m^2^) gave their informed written consent to participate in this study, which was approved by the ethical committee of the Human Anatomy Department, State University of Milan. Participants practiced for at least three hours/week (two training sessions) apart from the match day. All players were naturally right-footed. Leg preference was checked with the Waterloo Footedness Questionnaire – Revised (Elias et al., 1998): a score lower than 0.5 was registered for each participant, which indicates right-leg preference. All participants had been playing soccer for at least 5 seasons. During the experiment, players wore underwear and their own pair of indoor soccer shoes. The laboratory was equipped with an artificial turf carpet. After a standardized warm-up session of about 10 minutes (jogging, dribbling, short passes), each participant performed 30 inside-of-the-foot kicks (15 with the preferred and 15 with the non-preferred leg) kicking a stationary ball (5-size FIFA approved, mass: 422 g) towards a small football goal, placed 7 m far. This distance is comparable with a short-pass in a real game situation (O’Reilly and Wong, 2012). Players were free to choose where to start their approach run, standing still in a semicircular area with a radius of 1.5 m behind the ball. The goal (1.2 m wide and 0.8 m high) was horizontally divided in three areas of 0.4 m each with a plastic tape. Players were instructed to kick as accurately as possible: only passes hitting the center of the target were considered. This was a sort of normalization of trials, *i.e*. we considered preferred and non-preferred leg shots with the same (accurate) outcome. The distribution of accurate and inaccurate kicks was recorded for each foot. In order to reduce any learning effect, we administered the trials in alternating blocks of five passes with the preferred and five with the non-preferred leg.

### Testing apparatus

**Figure 1:**
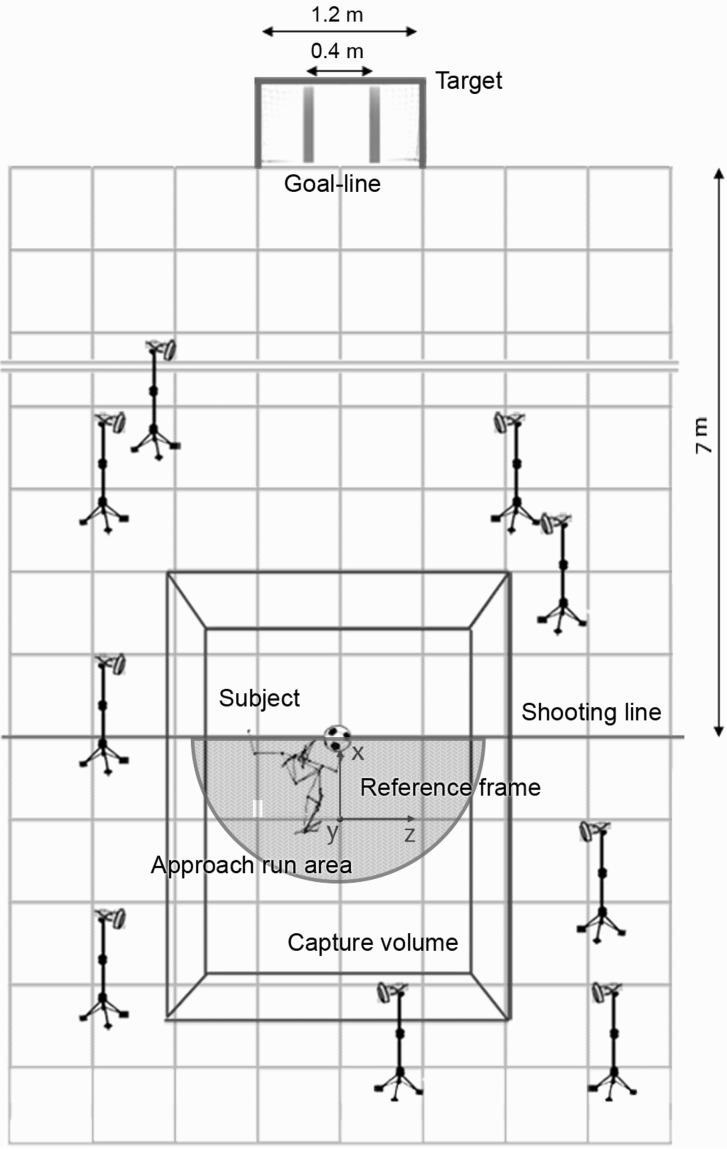
Laboratory setting: positioning of the subject, of the target and of the nine infra-red cameras

An optoelectronic motion analyzer (BTS S.p.A, Garbagnate Milanese, Italy) was used to acquire the movement of each subject. Nine infrared cameras were positioned around a working volume of 2.20 × 1.98 × 2.75 m (x, y, z), enough to capture movements from the last step of the approach run to the foot-ball impact (Figure 1). System calibration returned an average error of 0.59 ± 0.69 mm, with an accuracy (average error to volume diagonal ratio) of 0.015%. The system recorded (sampling rate: 120 Hz) the three-dimensional coordinates of 20 anatomical landmarks, identified by passive markers (diameter: 1.5 cm) attached to the skin. Among them, 14 were required for CoM kinematics estimation, following the protocol validated by Mapelli et al. (2014): tragi, acromia, olecranons, radius styloid processes, greater trochanters, femoral lateral epicondyles, lateral malleoli. The CoM coordinates estimate proved to be as accurate as that yielded by the ground reaction forces method, considered as a reference, with a root means square error during common exercises lower than 20 mm. Markers 15–20 were applied on each shoe in correspondence to the heel, first and fifth metatarsal head, and 3 additional markers were fixed on the ball, to locate the instants of support leg heel-strike and foot-ball impact. Before the trials, each participant was acquired for a few seconds standing in the anatomical position, setting a reference for the anatomical angles computation.

### CoM and kinematics calculation

According to Katis et al. (2013), the largest contribution to soccer kick performance takes place during the last stages of the kick (backswing, forward motion and ball-impact phases). Thus, as already performed by Levanon and Dapena (1998), we concentrated on the ground-support phase (kick duration), which is the time span between the landing of the support heel and the impact of the kicking foot with the ball. To estimate body CoM coordinates we adopted the Segmental Centroid Method, which assumes the body anatomical structure as a collection of rigid bodies. According to the Whittset’s segmental human model (Chandler and Clauser, 1975), we considered the following segments: a head-neck complex, a torso, upper arms, lower arms, thighs; the shank and foot segments were combined as one segment (lower leg). Hands were not considered since their mass (0.6% of the overall body mass) is negligible. Anthropometric data, including segments’ mass distribution and CoM location, were taken from Winter (1990). Segmental inertial parameters allowed the computation of the body center of mass through the weighted average of the CoM of each segment:

$${\text{r}}_{\text{CoM}}=\left[{\text{x}}_{\text{CoM}}{\text{y}}_{\text{CoM}}{\text{z}}_{\text{CoM}}\right]=\sum_{\text{i}=1}^{\text{N}}\frac{{\text{m}}_{1}\cdot {\text{r}}_{1}}{\text{M}}$$

where

$$r_{\mathrm{CoM}}=\left[x_{t}y_{t}z_{t}\right]$$

are the CoM coordinates of the *i*-th body segment, *m*_i_ its mass and *M* the overall body mass. The 3D coordinates were expressed as a right-handed orthogonal reference frame fixed on the ground, with the following sign convention: *x* was horizontal and pointed to the center of the target (anteroposterior direction, AP), *y* was vertical and pointed upwards (craniocaudal direction, CC); *z* was perpendicular to *x* and *y* (mediolateral direction, ML). Each marker coordinates were filtered with a 10 Hz low-pass 2^nd^ order Butterworth filter.

To compare results between subjects, total-body CoM height was normalized to subjects’ body height. CoM displacements (total and resolved on the three planes) were computed by subtracting the value of CoM position at support foot heel-strike to that at foot-ball impact. These events were manually identified by visually inspecting marker trajectories using the motion capture software. Velocity and acceleration of the CoM 3D-path and related components were obtained through numerical differentiation. To draw time curves of each variable, tracks were time normalized and ensemble averages were computed for preferred and non-preferred leg kicks. The CoM-forearm distance was the length of the vector between the total-body CoM and the CoM of the forearm segment.

We introduced a simplified one degree-of-freedom (DoF) model for knee and elbow to get a general description of the behavior of these joints. Knee angles were defined by greater trochanters, femoral lateral epicondyles and lateral malleoli markers. Elbow angles were defined by: acromia, olecranons, radius styloid processes. Shoulders obliquity was the angle on the frontal plane formed by the vector connecting acromia with the *z*-axis of the global reference. This angle is 0° if the shoulder segment is horizontal, and positive when the trunk is leaning on the kicking side. Shoulders rotation with respect to the goal line was the angle on the transverse plane between the vector connecting the acromia and a vector parallel to the goal (target) line. This angle is null if the shoulders segment is parallel to the target. Positive values underpin a rotation on the support limb, clockwise (right-foot kicks) or counterclockwise (left-foot kicks). Body angles (degrees), were assessed as offset from their values in standing position (e.g., extended legs refer to 0° knee flexion), with an estimated accuracy of 1 degree (Mapelli et al., 2013).

### Statistical analysis

An a priori power analysis was conducted over three relevant variables (CoM height and horizontal displacement, knee flexion angle) based on a previous pilot experiment. For an alpha (probability of type I error) of 0.05 and an effect size of 0.5, eight participants would give power of 0.8. The comparison between side-dependent kick accuracy was made by the Wilcoxon signed-rank test. For all the other variables, considering only the accurate shots, paired t-tests between preferred and non-preferred kick values were conducted. The Cohen’s *d* effect size coefficient for paired samples was calculated to determine if the statistical differences were practically significant. An effect size smaller than 0.3 was considered a “small” effect, around 0.5 a “medium” effect, larger than 0.8, a “large” effect (Cohen, 1992).

For figures and tables representation, the overall inter-subjects means and standard deviations were calculated. For all analyses, the significance level was set at 5% (*p*<0.05).

## Results

The success rate for the preferred-leg kicks was 45% and for the non-preferred leg kicks 33%. The results presented in Table 1 indicated non-significant differences in the duration between kicking sides (*p*>0.05), being around 150 ms in both conditions.

### CoM-related variables

**Figure 2:**
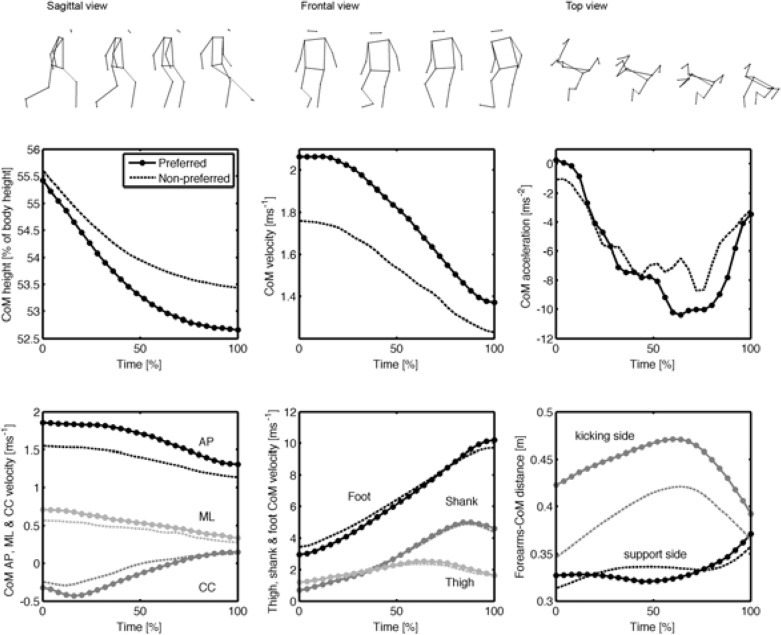
Time curves during the ground-support-phase (0% corresponds to support-foot heel-strike and 100% to foot-ball impact) of Center-of-Mass (CoM) height, CoM velocity, CoM acceleration (top), CoM velocity resolved in the three planes, lower limb segments’ CoM velocities and distance between forearms and body-CoM (bottom). Curves represent the means of resampled acquisitions of nine participants, the continuous line referring to preferred-leg and the dashed line to non-preferred leg kicks. AP stands for anteroposterior direction, ML for mediolateral and CC for craniocaudal. Sample kinetograms on the sagittal (left), frontal (center) and transverse plane are reported over the plots

Time averages of CoM-related parameters computed over all trials, divided by a kicking side, are reported in Figure 2. Normalized CoM height decreased during the kick, the non-preferred leg track being higher at all times and significantly different at foot-ball impact (*p*<0.001) between sides, with a large effect size (*d*=2.1). Average CoM displacements along the three planes were significantly higher when kicking with the preferred leg (*p*<0.001), as well as CoM velocity (*p*<0.01), precisely in the sagittal and coronal planes (*p*<0.01); large effect sizes were observed. No significant differences were found at impact for CoM CC velocity and CoM acceleration (*p*>0.05).

### Lower limbs kinematics

**Figure 3:**
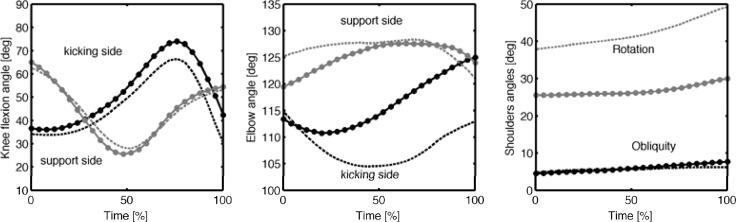
Time curves during the ground-support-phase (0% corresponds to support-foot heel-strike and 100% to foot-ball impact) of selected kinematic variables: kick- and support-side knee angles, elbow angles, shoulders obliquity. Curves represent the means of resampled acquisitions of nine participants, the continuous line referring to preferred-leg and the dashed line to non-preferred leg kicks.

Mean values of kinematic variables at foot-ball impact are presented in Table 2. Shank, thigh and foot velocities of the kicking leg exhibited delayed time curves, highlighting a proximal-to-distal motion pattern. At foot-ball impact, kicking side thigh velocity was similar in the two conditions, while kicking shank velocity resulted in about 10% higher (*p*<0.01), with a medium effect size (*d*=0.6). Foot velocity during the ground-support phase increased constantly up to approximately 10 m/s, resulting significantly higher at impact when kicking with the preferred leg (*p*<0.01), with an effect size of 1.7. No significant differences at impact were found regarding the support knee angle, whereas while kicking with the preferred leg the knee was significantly more extended (*p*<0.001) as compared to kicking with the non-preferred leg. Time curves, reported in Figure 3, show a similar trend in both cases.

### Upper body kinematics

Distance between the support-side forearm and total-body CoM was wider with the preferred leg during all the ground-support phases (Figure 2), and significantly different (*p*<0.01) at impact. The forearm velocity was significantly lower in preferred leg kicks on both the support side (*p*<0.05) and the kicking side (*p*<0.01), with a large effect size. The support-side elbow flexion/extension angle (Figure 3), did not differ at impact with respect to kicking laterality (small effect size). The shoulders inclination towards the target (*i.e.* upper trunk orientation) was lower when kicking with the preferred leg (*p*<0.01, large effect size), while shoulders obliquity relative to the ground was higher (*p<*0.01, *d*=1.3).

## Discussion

The main finding of this study was that, in non-professional adult players, there were dominance-related differences in kinematics when performing a precise inside-of-the-foot kick. In particular, when kicking with the preferred leg, CoM displacements and CoM velocity were higher (1), CoM was kept lower (2), the knee was more extended (3), and different arrangements of shoulders and arms (4) were observed.

When kicking with their preferred leg, subjects were more accurate than when kicking with their non-preferred leg. In contrast, Nunome et al. (2006) found that elite soccer players, whether right- or left-footed, were capable of delivering adequately fast and accurate passes with both feet. They stated that differences between sides in kick biomechanics depended on the skill level of the players. It could then be hypothesized that it was because of the amateur level of players analyzed in the current study that we could observe laterality differences in the inside-of-the-foot kick kinematics.

Ground-support phase durations were about 150 ms and not different between sides. This was already observed by McLean and Tumilty (1993) and Kawamoto et al. (2007), who showed no significant difference in the total time of kicking between an experienced (non professional players with 10–15 years of practice) and an inexperienced group. Katis et al. (2013) presented comparable values, while Levanon and Dapena (1998) found slightly lower values in highly experienced players. We can argue that the duration of the ground-support phase is not a relevant parameter in distinguishing the level or the laterality of players. Kellis et al. (2004) measured 170–190 ms between foot-landing and foot-ball impact in the instep kick. Higher values were due to the longer kicking leg backswing phase required to perform a more powerful shot.

### Body CoM

According to the Bernstein’s theory of the skill acquisition process, a learner initially demonstrates rigid and awkward coordination mode, known as “freezing” joint motion (Chow, 2007). In other words, novel complex motor skills are initially approximated by “freezing” degrees of freedom. Subsequently, higher stages of skill proficiency are characterized by a more differentiated use of DoF, and joint motion is gradually freed. When kicking with the preferred leg, CoM displacements along every direction were significantly higher and faster. Thus, there was a reduction of CoM movement when kicking with the non-preferred leg, with the same kick outcome. This is well explained by the Bernstein’s theory: non-preferred leg kicking is naturally less trained than preferred leg kicking. Therefore, global movement is somehow more rigidly controlled, and as few as possible of the involved body segments are controlled independently. This may be a way of reducing coordinative complexity at the sake of a poorer (slower, in this case) performance. This will be evident when considering upper body movement during the kick. Clearly, from the CoM perspective we are assessing only the overall effect of motor control on each single limb, which will be discussed in the following paragraphs.

During the ground-support phase, the body globally decelerates and CoM CC displacement is negative, so the body CoM is gradually lowered. These might be the effect of a motor strategy that ensures stability while hitting the ball. Moreover, normalized CoM height was significantly lower when kicking with the preferred leg. McCollum and Leen (1989) stated that the lower the body CoM was kept during a task, the higher the chance of maintaining stability. This is particularly important in martial arts, where balance control represents the key determinants of performance (Filingeri et al., 2012), and the strategies adopted to gain better stability are ankle/hip manoeuvres like lower limbs flexion with consequent lowering of the body CoM.

For this reason, and since participants displayed significant accuracy differences in the two conditions, we hypothesized that our players were more stable when kicking on the more trained (preferred) side. This is congruent with the Bernstein’s theory (higher CoM CC displacement at a higher skill level), and with Fletcher and Long (2013) observations: they studied professional soccer players’ balance skills while kicking with both legs and they recorded significantly worse dynamic balance when the dominant leg was used for stabilization tasks (i.e. non-preferred leg kicking).

### Lower limbs

When the support-foot landed, the support-side knee was flexed with an angle of about 65°. The shank was progressively flexed, getting to an angle of 25°–30° at the half of the ground-support phase. Subsequently, the shank was extended again, resulting in a knee angle of about 50°. Similarly, Lees et al. (2010) reported a support-knee angle of 45°. The kicking knee followed an inverse pattern, being extended up to the 80% of the support-phase, then rapidly flexed to an angle of 42° (preferred leg) and 30° (non-preferred leg) at foot-ball impact. It is well known that a kicking limb follows a proximal-to-distal motion pattern (Kellis and Katis, 2007; Nunome et al., 2006). This explains the behavior of thigh, shank and foot velocities curves, that appear to be delayed one to each other and increased in magnitude. Most of the speed of the foot is generated through knee extension (Levanon and Dapena, 1998), and the more the kicking leg is extended at impact, the higher the foot velocity (Lees et al., 2010). Our results support this finding: we found that the kicking-side knee was significantly more extended at ball-impact, and we recorded higher shank and foot velocities in preferred-leg kicks, which is consistent with the observations of Dörge et al. (2002) and Nunome et al. (2006). Levanon and Dapena (1998) reported a foot velocity of 8.4 m/s and Kawamoto et al. (2007) of 11.5 m/s, highlighting also (alongside Shan and Westerhoff, 2005) that the knee range of motion was higher in the experienced group. The explanation of this asymmetry may lie in the so-called ‘speed-accuracy trade-off’ (Kellis and Katis, 2007; Russell et al., 2010). Many studies on the instep-kick described the quality of the kick in terms of ball speed (Kawamoto et al., 2007; Kellis et al., 2004; Shan and Westerhoff, 2005), which is an important biomechanical indicator of success. However, it should be considered that also precision is a critical variable in a game situation (Russell et al., 2010). This is particularly true for the inside-of-the-foot kick, used for short and precise passes or shots. In the laboratory environment, the presence of a target determined the constraints on precision, leading to a trade-off between speed and accuracy. When players kicked with the non-preferred leg, a lower knee extension was observed (and consequently lower foot speed), toghether with a lower CoM displacement and velocity. This evidence might be the effect of a precise motor strategy: the neuromotor system reduced the execution speed on the less-trained side in order to maintain desired accuracy.

### Upper body

Upper body contribution to kick performance received little interest in literature. Shan and Westerhoff (2005) concentrated on the instep-kick: they noticed that right and left upper limbs behaved asymmetrically during the kick. They also noticed that slight asymmetry observed in a novice group was more noticeable in experienced players. Our results confirm this finding: though not statistically significant, we observed that when kicking with the non-preferred leg, the support-side forearm was about 40 mm closer to the body CoM. These considerations suggest that the coordination of upper limbs can contribute to executing a kick at a higher skill level. When kicking with the preferred leg, the support-side forearm was considerably more distant (13% at impact) to the trunk than when kicking with the non-preferred leg. At the same time, both elbows were extended in a range between 110°-125° (Figure 2). It follows that, when kicking with the preferred leg, the non-kicking side shoulder was more abducted than when kicking with the non-preferred leg. It has been suggested that the non-kicking side arm plays a role in the kick biomechanics: a skilled player will use trunk rotation and arm extension and abduction on the support-side to form a “tension arc” at the beginning of the kick step (Shan and Westerhoff, 2005). The inside-of-the-foot kick, however, requires less power than the instep-kick, so the arm elevation may be attributed to the maintenance of stability, which appears to be better when kicking with the preferred leg. Accordingly with the Bernstein’s theory, with practice, skilled participants (preferred-leg kickers) show less rigidity in their coordination pattern (Egan et al., 2007), “freeing” upper limbs DoFs.

Shoulders rotation with respect to the target was significantly lower when kicking with the preferred leg, meaning that players were “facing” the goal more directly. That is in line with the trunk rotation towards the kick side during the release of the tension arc discussed by Shan and Westerhoff (2005) and can be a valuable instruction for players in the learning process. Although we did not measure directly shoulder kinematics, we can suppose there is a relationship between trunk rotation, support-side arm abduction and horizontal extension, which could be an interesting issue for future surveys.

## Conclusion

The level of players involved in this study does not allow drawing general conclusions about laterality-related kinematics differences in the pass kick. Thus, results may be taken as preliminary insights into this issue; a larger (possibly elite or sub-elite) group should be considered for further research. The adaptation in passing a moving ball, as often happens in the game, or the angle and velocity of the approach run (which has been proven to influence the kick performance (Kellis et al., 2004)), may also be taken into account. In the investigated group our analysis outlined some differences between the preferred and non-preferred leg kicks. We considered only accurate shots, so that only distinctions due to laterality could emerge.

The main differences we found were as follows: higher CoM displacements and velocity, as well as lower normalized CoM height when kicking with the preferred leg; higher foot and shank velocities when kicking with the preferred leg; different positioning in space and velocities of arms, which were more abducted when kicking with the preferred leg, and shoulders, that were directed more towards the target in preferred leg kicking.

Coaches should consider the last finding while instructing young players, since “rotate shoulders towards the target” and “get quickly on the ball” may be simple and effective instructions. The adopted approach allowed the assessment of the kinematics of a sport skill while maintaining a perspective on the complete picture of movement.

## Figures and Tables

**Table 1:** Global and Center-of-Mass (CoM)-related variables at foot-ball impact. Comparison between kicks performed with the preferred and the non-preferred leg. AP stands for anteroposterior direction, ML for mediolateral and CC for craniocaudal. When not otherwise specified, between sides t-test p-values are reported

	Preferred leg (m±SD)	Non-preferred leg (m±SD)	p	Effect Size
**Global data**				
Accuracy [%]	45.2±8.7	32.6±11.8	< 0.01^#^	-
Duration [s]	0.16±0.03	0.15±0.04	0.519	0.1
**Body CoM parameters**				
CoM height [%]	52.7±0.9	53.4±1.0	< 0.001	2.1
Total CoM displacement [mm]	258±49.0	225±43.9	< 0.001	3.5
CoM AP displacement [mm]	231±42.4	209±38.1	0.001	1.8
CoM ML displacement [mm]	92.7±38.3	62.4±34.6	< 0.001	2.3
CoM CC displacement [mm]	−51.3±17.4	−37.4±10.8	< 0.001	3.3
CoM linear velocity [m/s]	1.37±0.31	1.23±0.25	0.003	1.5
CoM AP velocity [m/s]	1.31±0.26	1.14±0.33	0.002	1.7
CoM ML velocity [m/s]	0.37±0.14	0.25±0.15	0.004	1.4
CoM CC velocity [m/s]	0.15±0.09	0.13±0.08	0.17	0.6
CoM linear acceleration [m/s^2^]	−3.45±1.95	−3.15±2.09	0.30	0.5

^#^ : Wilcoxon signed-rank test

**Table 2:** Selected segmental Center-of-Mass (CoM) and anatomical angles at foot-ball impact. Comparison between kicks performed with the preferred and the non-preferred leg. AP stands for anteroposterior direction, ML for mediolateral and CC for craniocaudal. Between sides t-test p-values are reported

	Preferred leg (m±SD)	Non-preferred leg (m±SD)	p	Effect Size
**Segmental CoM parameters**				
CoM-forearm distance (kicking side) [mm]	401±73.1	389±89.6	0.531	0.2
CoM-forearm distance (support side) [mm]	404±48.0	348±51.5	0.004	1.5
Forearm velocity (kicking side) [m/s]	0.75±0.50	1.22±0.37	< 0.001	2.6
Forearm velocity (support side) [m/s]	2.66±0.59	3.00±0.99	0.419	0.9
Kicking thigh AP velocity [m/s]	1.64±0.39	1.75±0.09	0.060	0.8
Kicking shank AP velocity [m/s]	4.91±1.18	4.62±0.99	0.002	0.6
Kicking foot AP velocity [m/s]	10.2±0.65	9.72±0.88	0.002	1.7
**Anatomical angles**				
Knee, support-side [deg]	54.5±6.6	53.2±6.3	0.441	0.3
Knee, kicking-side [deg]	42.4±13.0	30.4±14.9	< 0.001	1.9
Elbow, support-side [deg]	124±10.8	121±11.5	0.482	0.2
Elbow, kicking-side [deg]	125±5.8	113±5.7	0.359	0.3
Shoulders obliquity [deg]	7.7±3.3	6.2±3.6	0.006	1.3
Shoulders rotation [deg]	30.0±8.9	36.6±8.7	0.004	1.5
